# Modulating Emission of Boric Acid into Highly Efficient and Color‐Tunable Afterglow via Dehydration‐Induced Through‐Space Conjugation

**DOI:** 10.1002/advs.202300139

**Published:** 2023-03-22

**Authors:** Zhen Zhang, Zhenguang Wang, Xiao Liu, Yu‐e Shi, Zhiqiang Li, Yanli Zhao

**Affiliations:** ^1^ Key Laboratory of Chemical Biology of Hebei Province Key Laboratory of Medicinal Chemistry and Molecular Diagnosis Ministry of Education College of Chemistry and Materials Science Hebei University Baoding 071002 China; ^2^ Tianjin Key Laboratory of Chemical Process Safety School of Chemical Engineering and Technology Hebei University of Technology Guangrong Dao 8, Hongqiao District Tianjin 300130 P. R. China; ^3^ School of Chemistry, Chemical Engineering and Biotechnology Nanyang Technological University 21 Nanyang Link Singapore 637371 Singapore

**Keywords:** afterglow, boric acid, room‐temperature phosphorescence, thermally activated delayed fluorescence, through‐space conjugation

## Abstract

Inorganic boric acid (BA) is generally not considered an efficient afterglow material, and several groups have reported its extremely weak room‐temperature phosphorescence (RTP) in the blue spectral region. It is discovered that heat treatment of BA results in increased afterglow intensity (27‐fold increase) and prolonged emission lifetime (from 0.83 to 1.59 s), attributed to enhanced through‐space conjugation (TSC) of BA. The afterglow intensity of BA can be increased further (≈415 folds) by introducing p‐hydroxybenzoic acid (PHA), which contains a conjugated molecular motif, to further promote the TSC of the BA system. This combination results in the production of afterglow materials with a photoluminescence quantum yield of 83.8% and an emission lifetime of 2.01 s. In addition, a tunable multicolor afterglow in the 420–490 nm range is achieved owing to the enhancement of the RTP and thermally activated delayed fluorescence of PHA, where BA exerts a confinement effect on the guest molecules. Thus, this study demonstrates promising afterglow materials produced from extremely abundant and simple precursor materials for various applications.

## Graphical abstract

By excluding the contribution of impurities to the afterglow of boric acid, the introduction of *p*‐hydroxybenzoic acid into the boric acid system leads to a 415‐fold increase in the afterglow intensity along with enhanced emission quantum yield of 83.8% and emission lifetime of 2.01 s.

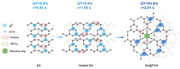

## 1 Introduction

After glow is a fascinating optical phenomenon, whereby light emission persists for a long time after the excitation source is turned off.^[^ ^1^, ^2^ ^]^ It has attracted considerable attention because of its excellent application potential in various fields, such as information encryption, bioimaging, and optoelectronics.^[^ ^3^, ^4^ ^]^ Conventional afterglow materials are dominated by inorganic materials doped with rare earth or transition metal ions, or organometallic systems relying on noble metals such as iridium and platinum.^[^ ^2^, ^5^ ^]^ Recent research endeavors have been focused on developing pure organic materials with long‐lived room‐temperature phosphorescence (RTP) or thermally activated delayed fluorescence (TADF).^[^ ^4^, ^6^, ^7^ ^]^ However, their emissions are still less efficient owing to the inherent inefficiency due to intersystem crossing from the lowest excited singlet state to the triplet manifold and the rapid annihilation of the triplet state by quenchers and fast nonradiative relaxation processes.^[^ ^2^, ^8^ ^]^ Thus, it is of great significance to develop highly efficient metal‐free afterglow materials with simple structures through facile and straightforward preparation processes.

Boric acid (BA), which is structurally simple and has an empty *p*‐orbital and electron‐accepting capability, is a versatile structural motif for fabricating afterglow materials.^[^ ^9^, ^10^ ^]^ BA can also be used as a rigid matrix to inhibit the molecular motion‐assisted nonradiative decay process, which is promising for developing materials that exhibit delayed fluorescence and phosphorescence.^[^ ^11^, ^12^, ^13^ ^]^ In addition, BA has recently been reported to exhibit blue RTP. However, the afterglow from BA is rather weak, and the photoluminescence (PL) quantum yield (QY) is as low as 0.82%.^[^ ^14^ ^]^ The emission color of BA is limited to the blue region, and the origin of its afterglow is still under debate. Wu et al. reported that BA with an extremely high purity of 99.99% exhibited RTP and attributed the blue afterglow of BA to through‐space conjugation (TSC) between the unpaired electrons of the O atoms in the B‐O confined space.^[^ ^14^ ^]^ On the other hand, Marder et al. attributed the blue RTP to impurities in BA by comparing the spectra of commercial and lab‐synthesized BA.^[^ ^15^ ^]^ However, the composition and photophysical properties of the impurity have not been clearly illustrated. The above mechanisms are still difficult to validate because it is extremely difficult to synthesize BA with a purity higher than 99.99%, and trace impurities may quench or enhance the RTP of BA. Thus, it is highly desirable to elucidate the mechanism underlying the RTP of BA. Moreover, it is necessary to enhance its emission efficiency and extend the emission color range.

**Figure 1:**
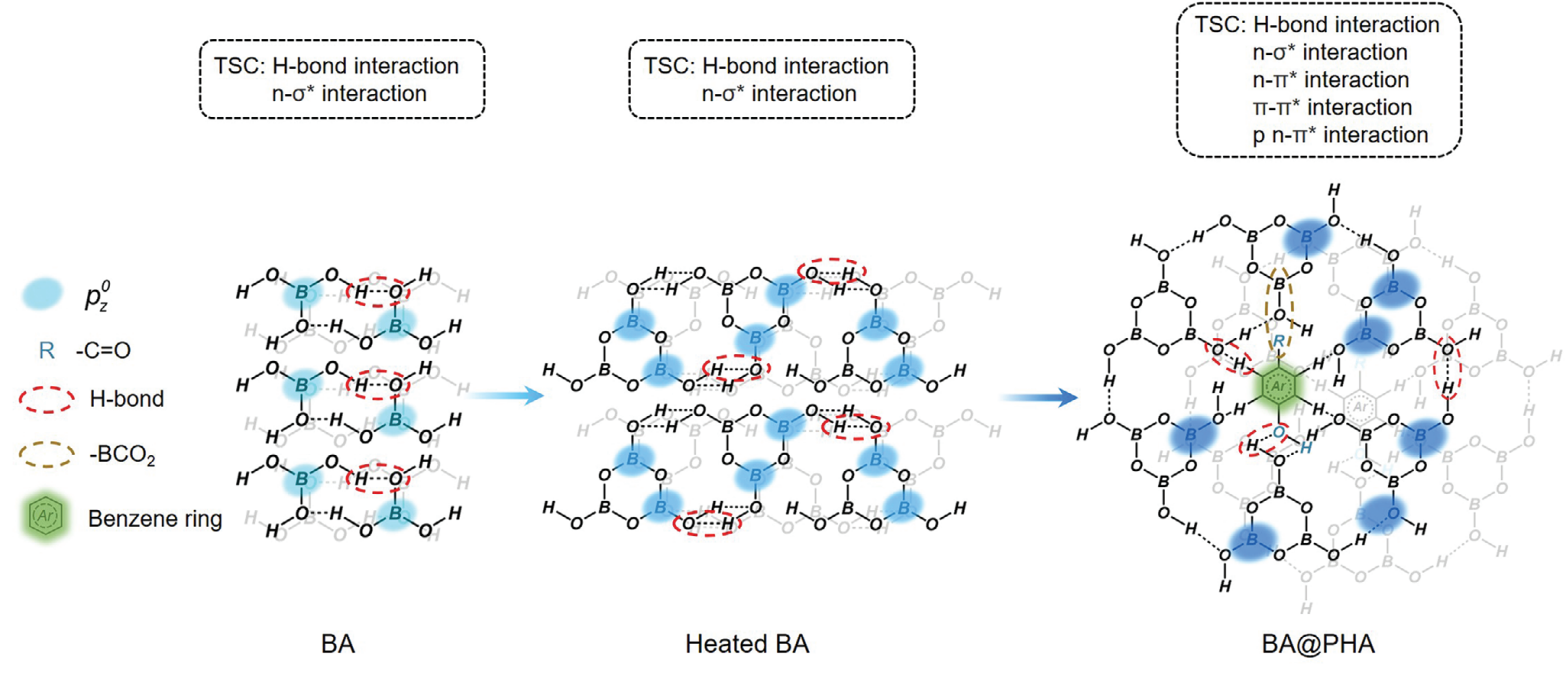
Schematic illustration of the heat treatment process and the increase of TSC in the system.

Herein, we demonstrate that the afterglow intensity and emission color range of BA can respectively be enhanced and extended through a straightforward heat‐treatment strategy. Following heat treatment, BA was transformed into a well‐packed mixture of metaboric acid and boric oxide, which resulted in the enhancement of the degree of TSC (**Figure** **1**), leading to a 27‐fold increase in the afterglow intensity, an increase in the PL QY from 0.8% to 5.9%, and the prolongation of the emission lifetime from 0.83 to 1.59 s. The afterglow performance was further improved by loading *p*‐hydroxybenzoic acid (PHA) into the BA system, thus achieving a 415‐fold increase in the afterglow intensity, a high PL QY of 83.8%, and an emission lifetime of 2.01 s. The loaded PHA enhanced the TSC of the system and inhibited the molecular motions of heated BA (Figure 1), which was responsible for its outstanding afterglow performance. In addition, the heated BA also acted as a matrix for locking and stabilizing the triplet states of guest molecules, thereby confining molecular motions and activating the TADF process of the guest. These synergistic effects enabled a tunable afterglow color in the wavelength range of 420–490 nm, which was further used in multilevel information encryption.

## 2 Results and Discussion

### 2.1 Photophysical Properties of As‐Received and Purified BA Samples

**Figure 2:**
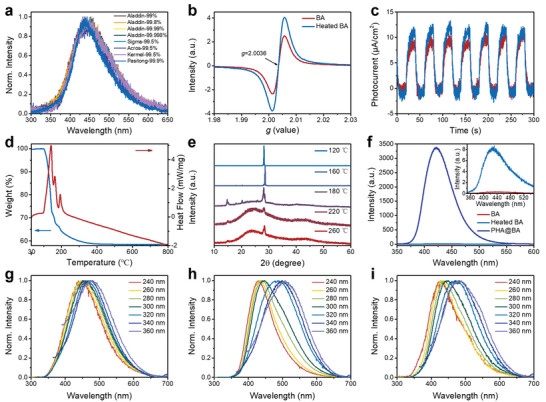
Photophysical properties of BA and related molecules. a) Normalized delayed PL spectra (excitation: 260 nm) of BA of different purities from different manufacturers. b) EPR spectra and c) current density as a function of the irradiation time for BA (red line) and heated BA (blue line). d) TGA and DSC curves of BA. e) Powder XRD patterns of BA after heat treatment at different temperatures. f) Delayed PL spectra of BA, heated BA, and BA incorporated with PHA; the enlarged spectra of BA and BA@PHA are shown in the inset. Normalized delayed PL spectra of g) BA, (h) metaboric acid, and (i) B_2_O_3_, under different excitation wavelengths.

First, we examined the delayed PL spectra of BA samples of different purities procured from different manufacturers; all the samples showed blue emission with a peak maximum located in the wavelength range of 437–446 nm (**Figure** **2**a). The BA samples were purified through recrystallization from both water and methanol solutions to remove possible inorganic and organic substances. The recrystallized BA was characterized by ^1^H nuclear magnetic resonance (NMR) and inductively coupled plasma mass spectrometry (Figure S1, Supporting Information). Figure S1a, Supporting Information, shows the ^1^H NMR spectrum of recrystallized BA, which presents one strong peak at 4.71 ppm. This spectrum confirms the absence of organic impurities in the recrystallized BA. As shown in Figure S1b, Supporting Information, the B atom is the main element after the inductively coupled plasma treatment, with an extremely trace and negligible amount of Ca^2+^. The purified samples showed almost the same blue delayed PL with the raw materials (Figure S2, Figure S3 and Table S1, Supporting Information), indicating that the contribution of impurities to the delayed PL of the BA can be ruled out. In addition, BA provided an electron paramagnetic resonance (EPR) signal at *g* = 2.0036 (Figure 2b) and a strong photocurrent signal in the photoconductivity curves (Figure 2c). These results indicate the presence of unpaired electrons in BA. Theoretically, no unpaired electrons exist in isolated BA, which has a vacant *p*_*z*_^0^ orbital, owing to the sp^2^ hybridization of B.^[^ ^10^, ^14^ ^]^ Thus, unpaired electrons should originate from packed BA. According to the structure of packed BA, there are overlaps among the *p* orbitals of B and O atoms, and the packed BA structure is further strengthened by intermolecular hydrogen bonding between neighboring molecules. The orbital overlaps allow the conjugation of molecules in space, that is, TSC, which may lead to the movement of electrons in the conjugated orbitals.^[^ ^16^ ^]^

To further confirm the TSC related to orbital conjugation, the structure and composition of packed BA was modulated through heat treatment, which resulted in the dehydration of BA to metaboric acid and then to boric oxide (B_2_O_3_).^[^ ^17^ ^]^ Thermal analysis by differential scanning calorimetry (DSC) and thermogravimetric analysis (TGA) suggested that the dehydration of BA to metaboric acid started at 98 °C (Figure 2d) and completed at ≈156 °C. The X‐ray diffraction (XRD) pattern of heated BA was also consistent with the results of thermal analysis, which suggested the formation of metaboric acid and B_2_O_3_ (Figure 2e).^[^ ^18^, ^19^ ^]^ According to the theory of TSC, the TSC of heated BA should be similar to or better than that of the starting material, and it should also exhibit delayed PL intensity.^[^ ^14^, ^16^ ^]^ As expected, the heated BA presented an enhanced EPR signal at *g* = 2.0036 (Figure 2b) and a higher photocurrent signal (Figure 2c). After heat treatment, a 27‐fold increase was observed in the delayed PL intensity of BA (Figure 2f, inset). The heat treatment resulted in the transformation of BA into a mixture of metaboric acid and B_2_O_3_ with a denser packing structure, which promoted the overlaps among the *p* orbitals of B and O atoms, as well as intermolecular hydrogen bonding. Consequently, an increase in the TSC, an increase in PL QY from 0.8 to 5.9% and the prolongation of the emission lifetime from 0.83 to 1.59 s were observed (Figure S4, Supporting Information). The TSC theory was further confirmed by the delayed PL spectra of metaboric acid and B_2_O_3_. The intensity of the delayed PL increased in the order of BA, metaboric acid, and B_2_O_3_ (Figure S5 and Table S2, Supporting Information). All three species showed blue delayed PL with excitation‐wavelength‐dependent emission properties (Figure 2g–i), which are typical features of molecules with TSC.^[^ ^20^ ^]^

### 2.2 Synthesis and Characterization of BA@PHA

**Figure 3:**
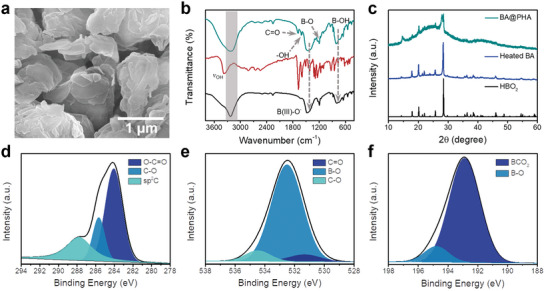
Characterization of BA@PHA. a) SEM image of BA@PHA. b) FTIR spectra of BA (black line), PHA (red line), and BA@PHA (cyan line). c) XRD patterns of heated BA (blue line), BA@PHA (cyan line), and simulated metaboric acid (black line). High‐resolution d) C1s, (e) O1s, and (f) B1s XPS profiles of BA@PHA.

Based on the obtained results, we inferred that introducing a more conjugated molecular motif could improve the degree of TSC of the BA system, which could in turn increase the emission QY and lifetime. Especially, molecules with benzene ring/carboxyl groups may react with metaboric acid and boron oxide in the matrix through polar–*π* interactions, which would be beneficial for improving the afterglow performance. Thus, *p*‐hydroxybenzoic acid (PHA), an aromatic compound with –OH and –COOH groups, was combined with BA through a straightforward heat‐treatment strategy.^[^ ^21^ ^]^ The resultant product is denoted as BA@PHA. In the scanning electron microscopy (SEM) images of BA@PHA (**Figure** **3**a), irregularly shaped particles with a diameter of ≈1 µm were observed. After heat treatment, PHA molecules were successfully incorporated in the matrix of BA, as confirmed by Fourier‐transform infrared (FTIR) and X‐ray photoelectron spectroscopic (XPS) measurements. The FTIR spectrum of BA@PHA clearly shows IR absorption bands at 3226, 1450, and 815 cm^−1^, corresponding to the stretching vibrations of OH and B–O bonds, and deformational vibrations of the B‐OH bond, respectively (Figure 3b).^[^ ^11^, ^22^ ^]^ The bands located at 1700 and 1600 cm^−1^ correspond to the carboxylic acid C=O and phenolic –OH of PHA.^[^ ^23^ ^]^ Compared with those of BA and PHA, the band corresponding to *v*_OH_ of BA@PHA in the wavenumber range of 3000–3200 cm^−1^ was broader and blue‐shifted. This trend was also observed in the Raman spectra (Figure S6, Supporting Information). These results suggest the formation and/or strengthening of H‐bonds in BA@PHA.

Furthermore, the XRD patterns of the heated BA and BA@PHA were analyzed to study the state of the BA matrix. The XRD patterns of both the heated BA and BA@PHA samples matched well with the simulated XRD pattern of metaboric acid (Figure 3c).^[^ ^9^, ^18^ ^]^ Moreover, the XRD peaks of BA@PHA were not as sharp as those of heated BA, which is a characteristic indication of the formation of B_2_O_3_.^[^ ^17^ ^]^ The TGA curve of heated BA shows that the weight almost keeps constant in the temperature range from 30 to 100 °C (Figure S7, Supporting Information), indicating the absence of water in heated BA sample. From the signal intensities of C, O and B in the full‐scan XPS profile of BA@PHA, their contents were determined to be 6.2, 51.3, and 42.5%, respectively (Figure S8, Supporting Information). The high‐resolution C1s XPS profile of BA@PHA could be fitted with three peaks at 287.8, 285.6, and 284.0 eV (Figure 3d), corresponding to C=C, C–O, and O–C=O linkages, respectively, which suggested the successful loading of PHA in BA.^[^ ^11^ ^]^ The high‐resolution O1s XPS profile (Figure 3e) was fitted with three peaks related to C=O (531.3 eV), B–O (532.4 eV), and C–O (534.1 eV).^[^ ^19^ ^]^ Further, the high‐resolution B1s profile (Figure 3f) was fitted with two peaks centered at 192.8 and 194.2 eV, attributable to boron atoms surrounded (BCO_2_) by carbon and oxygen atoms, and B−O bonds, respectively.^[^ ^12^ ^]^ These results suggest the successful loading of PHA in the BA matrix and also indicate that the BA matrix, which is composed of metaboric acid with partial formation of B_2_O_3_, is crosslinked by H‐bonds.

### 2.3 Photophysical Properties of BA@PHA

**Figure 4:**
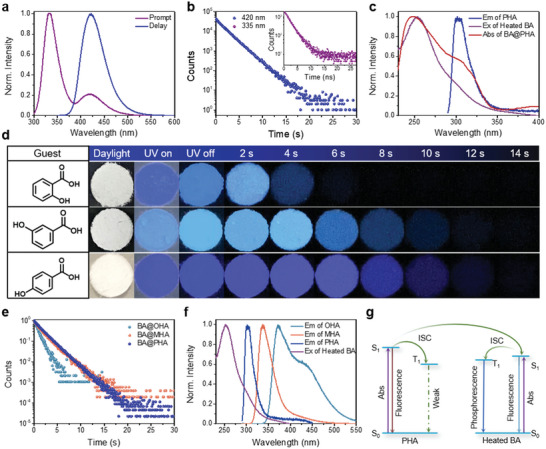
Delayed PL properties of BA@PHA and mechanism investigation of the afterglow. a) Prompt and delayed PL spectra of BA@PHA. b) Emission decay curves of BA@PHA were detected at 420 and 335 nm (excited at 280 nm). c) Prompt PL emission spectrum of PHA (blue line, excited at 280 nm), delayed PL excitation spectrum of heated BA (purple line, detected at 430 nm), and absorption spectrum of BA@PHA (red line). d) Digital photographs of afterglow materials were obtained by doping different guest molecules into BA under daylight, under UV light, and after different time intervals following the switching off of the UV light. e) Delayed PL decay curves of the afterglow materials obtained by doping different guest molecules into BA (excited and recorded at 280 and 430 nm, respectively). f) Normalized delayed PL excitation spectrum of heated BA (purple line, detected at 430 nm), and prompt PL emission spectra (excited at 280 nm) of OHA, MHA, and PHA. g) Simplified Jablonski diagram showing the energy transfer from PHA to heated BA (ET: energy transfer, ISC: intersystem crossing).

BA@PHA showed a deep‐blue emission under UV irradiation, and the blue emission could last more than 14 s after the excitation light was turned off (Video S1, Supporting Information). Two distinct peaks located at 335 and 420 nm were observed in the prompt PL spectrum (**Figure** **4**a), while the delayed PL spectrum exhibited a strong peak centered at 420 nm, which matched with the second peak of the prompt PL spectrum. To obtain a deeper insight into the emission features of BA@PHA, the decay curves of 335 and 420 nm emissions were recorded, from which their average emission lifetimes were determined to be 1.96 ns and 2.01 s, respectively (Figure 4b and Table S3, Supporting Information). Thus, the afterglow of BA@PHA was attributed to the delayed emission at 420 nm. The intensity and lifetime of the deep‐blue afterglow could be modulated by controlling the heat‐treatment conditions, including the heating temperature, pH, and loading amount of PHA (Figure S9 and Tables S4–S6, Supporting Information). In terms of the afterglow intensities and lifetimes of the products, the best performance was achieved upon loading 10 mg (0.07 mmol) of PHA into 3 g of BA by heating at 210 °C. The optimized conditions provided a BA@PHA sample with an ultralong lifetime of 2.01 s, a high PL QY of 83.8%, and an afterglow QY of 32.0%.

### 2.4 Mechanism for Ultralong and Efficient Afterglow of BA@PHA

To gain more insights into the photophysical properties of BA@PHA, the emission profiles of heated BA and BA@PHA were compared. The delayed PL spectrum of BA@PHA was almost identical to that for heated BA (Figure S10, Supporting Information), suggesting the same origin of their emission. The loading of PHA significantly enhanced the emission intensity (415‐fold increase) and extended the emission lifetime (from 1.59 s to 2.01 s) of heated BA. The enhancement of the emission intensity could be attributed to the increased degree of TSC and the confinement effect of heated BA on PHA. Upon forming BA@PHA, the more conjugated molecular motif of PHA interacted with BA via intermolecular hydrogen bonding, which increased the degree of TSC.^[^ ^9^, ^12^ ^]^ This was confirmed by photocurrent measurements, where an increased photocurrent signal was observed for BA@PHA (Figure S11, Supporting Information). The formation of BA@PHA also prevented the quenching of heated BA by many environmental parameters, such as O_2_, water, and other solvents exposed to BA. These effects led to the enhancement of the PL QY.

To further understand the origin of the prolonged emission lifetime, the photophysical properties of PHA were compared with those of BA@PHA. Pure PHA exhibited a strong PL peak at 300 nm and a rather weak delayed PL peak at 505 nm (Figure S12, Supporting Information). The prompt and delayed PL spectra of heated PHA samples were further measured, showing almost identical shape and peak maximum (Figure S12, Supporting Information). The prompt PL spectrum of PHA overlapped well with the delayed PL excitation spectrum of heated BA and the absorption spectrum of BA@PHA (Figure 4c). In view of the close association of the BA and PHA molecules in the solid state, the observed spectral overlap suggests the possibility of energy transfer from PHA to heated BA. After the formation of BA@PHA, the prompt PL of PHA was significantly decreased (Figure S13, Supporting Information), which confirmed our hypothesis and indicated a high energy‐transfer efficiency of the system.^[^ ^24^ ^]^ Aided by the energy‐transfer process, the emission lifetime was significantly improved. This result recalled that the afterglow intensity and lifetime of BA@PHA are related to the loading amount of PHA (Figure S9, Supporting Information), as explained by the difference in the efficiency of energy transfer when changing the molecular ratio of PHA and BA.

The energy transfer mechanism was further confirmed by control experiments using *o*‐hydroxybenzoic acid (OHA) and *m*‐hydroxybenzoic acid (MHA), which bear the same chemical groups as PHA at different positions on the benzene ring, as guest molecules to produce BA@OHA and BA@MHA, respectively. As shown in Figure 4d, BA@PHA, BA@OHA, and BA@MHA are light‐yellow powders that exhibit a blue afterglow. However, notable differences in the afterglow times (Figure 4d) and emission lifetimes (Figure 4e) were observed, with BA@PHA presenting the longest lifetime of 2.01 s and BA@OHA presenting the shortest lifetime of 0.73 s (Figure 4e and Table S7, Supporting Information). This can be explained by the degree of spectral overlap between the delayed PL excitation spectrum of heated BA and the prompt PL emission spectra of the guest molecules. As mentioned previously, a good overlap (>100 nm) occurred between the delayed PL excitation spectrum of heated BA and the prompt PL emission spectrum of PHA. However, a relatively narrow overlap and almost no overlap occurred for OHA and MHA, which resulted in less efficient energy transfer to extend the emission lifetime of the heated BA (Figure 4f). To better represent the energy‐transfer mechanism, a simplified Jablonski diagram is presented in Figure 4g.

### 2.5 Multiple Afterglow Colors of BA@PHA

**Figure 5:**
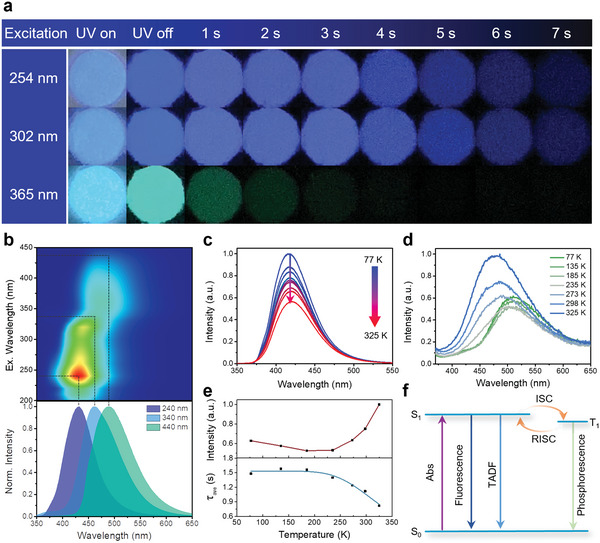
Mechanism investigation of the multiple afterglow colors in of BA@PHA. a) Digital photographs of BA@PHA under UV light and after different time intervals following the switching off of UV light (the sample was excited at different wavelengths). b) Excitation‐delay PL mapping of BA@PHA (top), and the delayed PL spectra obtained under different excitation wavelengths (bottom). Delayed PL spectra of BA@PHA recorded at different temperatures by exciting at c) 280 nm and d) 360 nm. e) Temperature‐dependent delayed PL intensity at different detection temperatures (top) and the plot of emission lifetime against the detection temperature for BA@PHA (bottom). f) Simplified Jablonski diagram for the emission of PHA.

Notably, BA@PHA produced by loading 100 mg (0.7 mmol) of PHA in 3 g of BA showed multiple afterglow colors under different excitation wavelengths (**Figure** **5**a). The emission color could be tuned from blue to green (in the wavelength range of 429–490 nm) by changing the excitation wavelength (Figure 5b). The afterglow spectra were asymmetrical, and the full‐width at half‐maximum values of the emissions were greater than 100 nm (Figure 5b). Significant differences were observed in the emission lifetimes measured at various excitation wavelengths (Figure S14 and Table S8, Supporting Information), and clear variations were also noted in the emission QY under different excitation wavelengths (Table S9, Supporting Information). These results strongly suggest the coexistence of multiple emissive species. To gain more insights into the emissive species, the temperature‐dependent delayed PL spectra of heated BA, pure PHA, and BA@PHA were recorded. Under 280 nm excitation, the intensity of the delayed PL of BA@PHA at 420 nm decreased with an increase in the detection temperature from 77 to 325 K, without a shift in the peak maximum (Figure 5c). This trend is similar to the temperature‐dependent spectral profile of the heated BA (Figure S15, Supporting Information). Upon changing the excitation wavelength to 360 nm, the delayed PL intensity of BA@PHA decreased with an increase in the detection temperature from 77 to 210 K, without the shift of the peak maximum at 510 nm. However, upon increasing the detection temperature to 235 K, a blue shift in the peak maximum to 498 nm was observed, and the peak shifted further with an increase in the detection temperature to 325 K (Figure 5d). This trend is a typical feature of TADF.^[^ ^25^ ^]^ In contrast, no shift in the peak maximum of the delayed PL was observed for pure PHA in the detection temperature range of 77 to 235 K, and only a rather weak peak was observed at 446 nm upon increasing the detection temperature further to 325 K (Figure S16, Supporting Information).

However, it seems inconsistent with previous results that the emission from a single state should be ≈300 nm (Figure 4f). To get further insight, the prompt PL spectra of pure PHA, excited at different wavelengths, were collected. As shown in Figure S17, Supporting Information, the peak maximum varies from 300 nm to 460 nm after changing the excitation wavelength from 260 to 360 nm. Thus, it was concluded that PHA shows excitation‐dependent emission peak maximum, and loading PHA into the matrix of heated BA could activate the TADF process. These results suggest that the afterglow of BA@PHA is the combined result of the delayed PL of heated BA and that of PHA. Being a conjugated molecular motif, PHA promoted the TSC of heated BA, leading to an enhanced delayed PL intensity in the deep‐blue region. The heated BA also acted as a matrix for the loaded PHA and exerted a confinement effect on PHA. It restricted the molecular motion of PHA, and thus decreased the probability of the nonradiative path, leading to enhanced emission intensity. In addition, heated BA also enhanced the TADF of PHA by providing a vacant *p* orbital. It attracted *π* electrons from PHA, forming p‐n‐π* conjugate systems, which are useful for reducing the energy of the minimum unoccupied orbital of PHA.^[^ ^11^ ^]^

To further confirm the above mechanisms, the emission decay curves of BA@PHA were collected at different temperatures (Figure S18 and Table S10, Supporting Information), and the curves of the emission lifetime versus detection temperature were fitted using Equation 1.^[^ ^26^ ^]^

(1) $$\tau_{\mathrm{ave}}=\frac{3+\exp\left(-\frac{\Delta E_{\mathrm{ST}}}{k_{B}T}\right)}{\frac{3}{\tau_{T}}+\frac{1}{\tau_{S}}\exp\left(-\frac{\Delta E_{\mathrm{ST}}}{k_{B}T}\right)}$$

where *τ*_T_ and *τ*_S_ are the fitted emission and decay lifetimes of the lowest triplet and singlet excited states, respectively, *T* is the detection temperature, *K*_B_ is the Boltzmann constant, and Δ*E*_ST_ is the energy gap between the *T*_1_ and *S*_1_ states. Through fitting analysis (Figure 5e), the Δ*E*_ST_ was calculated to be 0.1857 eV. This value is sufficiently small to meet the requirement for the reverse intersystem crossing process of TADF, which generally requires the Δ*E*_ST_ to be smaller than 0.2 eV.^[^ ^7^, ^8^ ^]^ For a better presenting these multiple emission species, the effects of RTP and TADF of PHA are schematically illustrated in Figure 5f.

### 2.6 Information Encryption Application of BA@PHA

**Figure 6:**
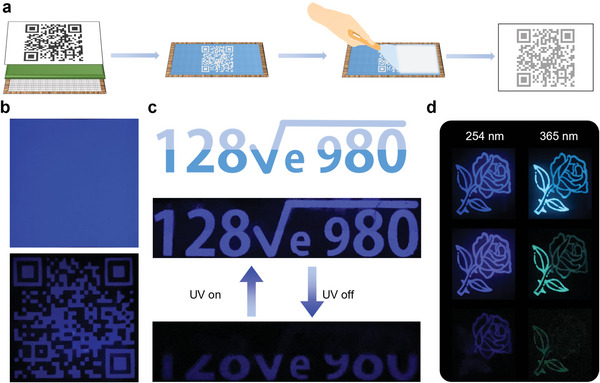
Information encryption applications of BA@PHA. a) Schematic illustration of the screen‐printing process. b) Digital photographs of the QR code of the official website of the College of Chemistry and Environmental Science of Hebei University on a piece of printing paper upon switching on (top) and switching off (bottom) 254 nm UV light. c) Patterns of a number and hidden code printed using BA@PHA samples produced under different pH upon switching on (top) and switching off (bottom) 254 nm UV light. d) Digital photographs of a flower pattern under the irradiation of 254 and 365 nm UV light, and after switching off the UV light for 2 s.

Given the high emission afterglow efficiency and tunable emission color of BA@PHA, its application in information encryption was evaluated. Different patterns and encryption codes were produced using the screen‐printing technology (**Figure** **6**a); BA@PHA was used as a solid ink. The QR code in the official website of the College of Chemistry and Environmental Science of Hebei University was printed on a piece of regular printing paper (Figure 6b). Under the irradiation of 365 nm UV light, the paper presented strong blue autofluorescence, with an emission color identical to that of BA@PHA. After switching off the UV light, the short‐lived fluorescence from the paper disappeared, and only the QR code was visible, which could be quickly identified (Video S2, Supporting Information).

In addition, patterns for anti‐counterfeit encryption‐related applications were designed using the difference in the emission lifetimes of the samples prepared under different pH conditions. As shown in Figure 6c, the solid ink in the gray part was composed of BA@PHA‐9.0, and that in the blue part was composed of BA@PHA‐3.0, which shows an intense blue emission under UV light irradiation. When the UV light was turned off, the emission of BA@PHA‐9.0 was invisible, while BA@PHA‐3.0 still presented a blue afterglow because of the difference in their emission lifetimes (0.73 s for BA@PHA‐9.0 versus 1.96 s for BA@PHA‐3.0). Thus, the pattern changed into “I Love You” after switching off the UV light for 5 s. The emission lifetime also depended on the loading amount of PHA. As shown in Figure 6d, the solid ink used to print the flower part was composed of BA@PHA‐10 mg while that used for the stem and leaves were composed of BA@PHA‐100 mg. Under 254 nm UV light, the pattern exhibited a strong blue emission. Upon switching off the UV light, an overall blue color was observed, and after 2 s, the stem and leaves became invisible. Under 365 nm UV light, the pattern exhibited a cyan emission. When the UV light was turned off, the overall color appeared light green, and after 2s, only the stem and leaves were visible (Video S3, Supporting Information).

## 3 Conclusion

Both the as‐received BA samples from different manufacturers with different purities and purified BA samples showed almost identical delayed PL behaviors, which excluded the contribution of impurities to the afterglow of BA. Heat treatment could modulate the structure and composition of inorganic BA, leading to a significant improvement in its afterglow intensity (∼27‐fold increase) and lifetime (from 0.83 to 1.59 s). Structural and photophysical investigations suggested that these improvements could be attributed to the enhanced degree of TSC among the B and O atoms. A 415‐fold increase in the afterglow intensity and prolonged emission lifetimes were achieved by introducing PHA into the BA system. A PL QY of 83.8% and an emission lifetime of 2.01 s were realized, which are attributed to the increase in TSC owing to the conjugated molecular motif. In addition, BA also acted as a matrix to modulate the photophysical properties of PHA and thereby strengthen the emission intensity and TADF process of PHA. These effects resulted in a tunable multicolor afterglow in the blue‐to‐green region. Aided by the high efficiency and color tunability of the afterglow features, the products were successfully utilized in multilevel information encryption. This study provides new insights to the emission mechanisms of BA and furnishes an example for studying and improving the afterglow properties of materials. The availability of efficient and color‐tunable afterglow materials could lead to their utilization in many fields such as solid lighting and displays.

## 4 Experimental Section

### Materials

PHA was supplied by Aladdin Chemicals. BA samples of different purities from different manufacturers were used, as presented in Table S1, Supporting Information.

### Recrystallization of BA

Typically, BA (6.2 g, 0.1 mol) suspended in 20 mL of H_2_O or methanol was heated at 60 °C until all the BA powder was completely dissolved. Then, the mixture was cooled, resulting in the precipitation of BA. Finally, the solid was separated from the solvent via filtration.

### Incorporation of PHA in BA

PHA was incorporated in BA using a heat‐treatment strategy. Typically, 3.0 g (48.5 mmol) of BA was dissolved in 40 mL of deionized water in a beaker and mixed with 0.01 g (0.07 mmol) of PHA. Then, the beaker was heated in an oven at 210°C for 3 h until a glassy solid was formed. A white powder (denoted as BA@PHA) was obtained by grinding the cooled product. Heated BA was obtained by heating an aqueous solution of BA (75 mg mL^−1^) at 210 °C for 3 h, followed by grinding the product obtained after cooling into a powder.

### Statistical Analysis

The differences between experimental groups were analyzed by Student's *t*‐test. The photoluminescence quantum yield of samples was measured and calculated from three parallel samples. All of the statistical analyses for the molecular spectra, XPS spectra, and XRD patterns were performed using the Origin software. Normalization of the photoluminescence spectra was carried out by setting the intensity at the peak maximum as 1. The data of emission lifetime were fitted using the exponential fitting function of Origin software, keeping the *χ* ^2^ in the range from 0.8 to 1.3.

## Conflict of Interest

The authors declare no conflict of interest.

## Supporting information

Supporting Information

Supplemental Video 1

Supplemental Video 2

Supplemental Video 3

## Data Availability

The data that support the findings of this study are available from the corresponding author upon reasonable request.

## References

[1] a) J. Guo , C. Yang , Y. Zhao , Acc. Chem. Res. 2022, 55, 1160; 35394748 10.1021/acs.accounts.2c00038

[2] W. Zhao , Z. He , B. Z. Tang , Nat. Rev. Mater. 2020, 5, 869.

[3] a) Y. Wang , H. Gao , J. Yang , M. Fang , D. Ding , B. Z. Tang , Z. Li , Adv. Mater. 2021, 33, 2007811; 10.1002/adma.202007811 33772942

[4] R. Gao , M. S. Kodaimati , D. Yan , Chem. Soc. Rev. 2021, 50, 5564. 33690765 10.1039/d0cs01463j

[5] a) L. Li , Z. Wu , P. Lv , C. Wang , X. Han , Y. Yang , Opt. Express 2022, 30, 31889; 36242262 10.1364/OE.459686

[6] a) T. Zhang , X. Ma , H. Wu , L. Zhu , Y. Zhao , H. Tian , Angew. Chem., Int. Ed. 2020, 59, 11206; 10.1002/anie.201915433 31876988

[7] X. Wang , Y. Sun , G. Wang , J. Li , X. Li , K. Zhang , Angew. Chem., Int. Ed. 2021, 60, 17138. 10.1002/anie.202105628 34060200

[8] Q. Peng , H. Ma , Z. Shuai , Acc. Chem. Res. 2021, 54, 940. 33347277 10.1021/acs.accounts.0c00556

[9] Z. Zhang , Y.‐e. Shi , Y. Liu , Y. Xing , D. Yi , Z. Wang , D. Yan , Chem. Eng. J. 2022, 442, 136179.

[10] M. Mutailipu , K. R. Poeppelmeier , S. Pan , Chem. Rev. 2021, 121, 1130. 33307685 10.1021/acs.chemrev.0c00796

[11] W. Li , W. Zhou , Z. Zhou , H. Zhang , X. Zhang , J. Zhuang , Y. Liu , B. Lei , C. Hu , Angew. Chem., Int. Ed. 2019, 58, 7278. 10.1002/anie.201814629 30924580

[12] X. Zheng , Y. Huang , W. Lv , J. Fan , Q. Ling , Z. Lin , Angew. Chem., Int. Ed. 2022, 61, e202207104. 10.1002/anie.202207104 35674723

[13] Y. Ding , X. Wang , M. Tang , H. Qiu , Adv. Sci. 2022, 9, 2103833. 10.1002/advs.202103833 PMC8787396 34799998

[14] H. Zheng , P. Cao , Y. Wang , X. Lu , P. Wu , Angew. Chem., Int. Ed. 2021, 60, 9500. 10.1002/anie.202101923 33594791

[15] Z. Wu , J. C. Roldao , F. Rauch , A. Friedrich , M. Ferger , F. Würthner , J. Gierschner , T. B. Marder , Angew. Chem., Int. Ed. 2022, 61, e202200599. 10.1002/anie.202200599 PMC9305524 35104020

[16] a) H. Zhang , Z. Zhao , P. R. McGonigal , R. Ye , S. Liu , J. W. Y. Lam , R. T. K. Kwok , W. Z. Yuan , J. Xie , A. L. Rogach , B. Z. Tang , Mater. Today 2020, 32, 275;

[17] C. Huber , S. S. Jahromy , F. Birkelbach , J. Weber , C. Jordan , M. Schreiner , M. Harasek , F. Winter , Energy Sci. Eng. 2020, 8, 1650.

[18] S. Balcı , N. A. Sezgi , E. Eren , Ind. Eng. Chem. Res. 2012, 51, 11091.

[19] S. Han , G. Lian , X. Zhang , Z. Cao , Q. Wang , D. Cui , C.‐P. Wong , Chem. Eng. J. 2021, 417, 129175.

[20] a) Y. Zhang , B. He , W. Luo , H. Peng , S. Chen , R. Hu , A. Qin , Z. Zhao , B. Z. Tang , J. Mater. Chem. C 2016, 4, 9316;

[21] a) J. Economy , R. S. Storm , V. I. Matkovich , S. G. Cottis , B. E. Nowak , J. Polym. Sci., Polym. Chem. Ed. 1976, 14, 2207;

[22] a) E. F. Medvedev , A. S. Komarevskaya , Glass Ceram. 2007, 64, 42;

[23] S. A. Brandán , F. Márquez López , M. Montejo , J. J. López González , A. Ben Altabef , Spectrochim. Acta, A 2010, 75, 1422. 10.1016/j.saa.2010.01.012 20223703

[24] S. Xu , W. Wang , H. Li , J. Zhang , R. Chen , S. Wang , C. Zheng , G. Xing , C. Song , W. Huang , Nat. Commun. 2020, 11, 4802. 32968080 10.1038/s41467-020-18572-9 PMC7511363

[25] a) W. Ma , Y. Su , Q. Zhang , C. Deng , L. Pasquali , W. Zhu , Y. Tian , P. Ran , Z. Chen , G. Yang , G. Liang , T. Liu , H. Zhu , P. Huang , H. Zhong , K. Wang , S. Peng , J. Xia , H. Liu , X. Liu , Y. M. Yang , Nat. Mater. 2022, 21, 210; 34764429 10.1038/s41563-021-01132-x

[26] Z.‐R. Yuan , Z. Wang , B.‐L. Han , C.‐K. Zhang , S.‐S. Zhang , Z.‐Y. Zhu , J.‐H. Yu , T.‐D. Li , Y.‐Z. Li , C.‐H. Tung , D. Sun , Angew. Chem., Int. Ed. 2022, 61, e202211628. 10.1002/anie.202211628 36104622

