# Supplementary material for: Modulating Emission of Boric Acid into Highly Efficient and Color‐Tunable Afterglow via Dehydration‐Induced Through‐Space Conjugation
Source: Adv Sci (Weinh). 2023 Mar 22;10(15):2300139. doi: 10.1002/advs.202300139 (PMC10214226; doi:10.1002/advs.202300139)
Supplement: Supplementary file 1 — Supporting Information [file ADVS-10-2300139-s003.pdf]

ADVANCED SCIENCE
Open Access

Supporting Information

for *Adv. Sci.*, DOI 10.1002/advs.202300139

Modulating Emission of Boric Acid into Highly Efficient and Color-Tunable Afterglow via Dehydration-Induced Through-Space Conjugation

Zhen Zhang, Zhenguang Wang, Xiao Liu, Yu-e Shi, Zhiqiang Li* and Yanli Zhao**

Supporting Information

1. Characterization

Prompt and delayed photoluminescence (PL) spectra (with a decay period of 25 ms) of samples were measured on a Hitachi F-7000 spectrometer. Fluorescence lifetime decay curves were recorded on an Edinburgh FS-5 spectrometer equipped with a xenon arc lamp (Xe900) or EPL laser at 280 nm. The afterglow lifetime was determined using the kinetic decay method on a Horiba FluoroMax+ spectrometer at the excitation wavelength of 280 nm. The absolute total PL quantum yield (QY) was measured on a Horiba FluoroMax+ spectrometer, equipped with an integral sphere, which included both fluorescence and phosphorescence emission components. The phosphorescence QY can be deduced and figured out through peak-differentiation-imitating analysis and by comparing the integrating area ratios of each component. UV-vis spectra were measured on a Shimadzu UV3600, in a reflectance mode using full reflection accessories, using barium sulphate as the reference. Fourier transform infrared (FTIR) spectra were measured on a Thermo Fisher Nicolet iS10 through KBr pellet method. Raman spectra were measured on a Horiba LabRAM HR Evolution Raman spectroscopy system equipped with a 532 nm diode laser source. Electron paramagnetic resonance (EPR) spectra were measured on a Bruker A300 EPR spectrometer. Powder X-ray diffraction (XRD) patterns were measured using a Bruker D8 Advance diffractometer (Cu Kα: λ = 1.5418 Å) under ambient conditions. The photocurrent responses were recorded over a sampling interval of 20 s on a Perfectlight PLS-LED 100C (60W white LED lamp). The SEM images were taken on a Zeiss Sigma 300. X-ray electron spectroscopy (XPS) spectra were recorded on a Thermo Scientific Escalab 250Xi+. TGA and DSC curves were measured on a Netzsch STA 449F3. The photographs and videos were taken by an iPhone 11 under ambient conditions.

2. Figures

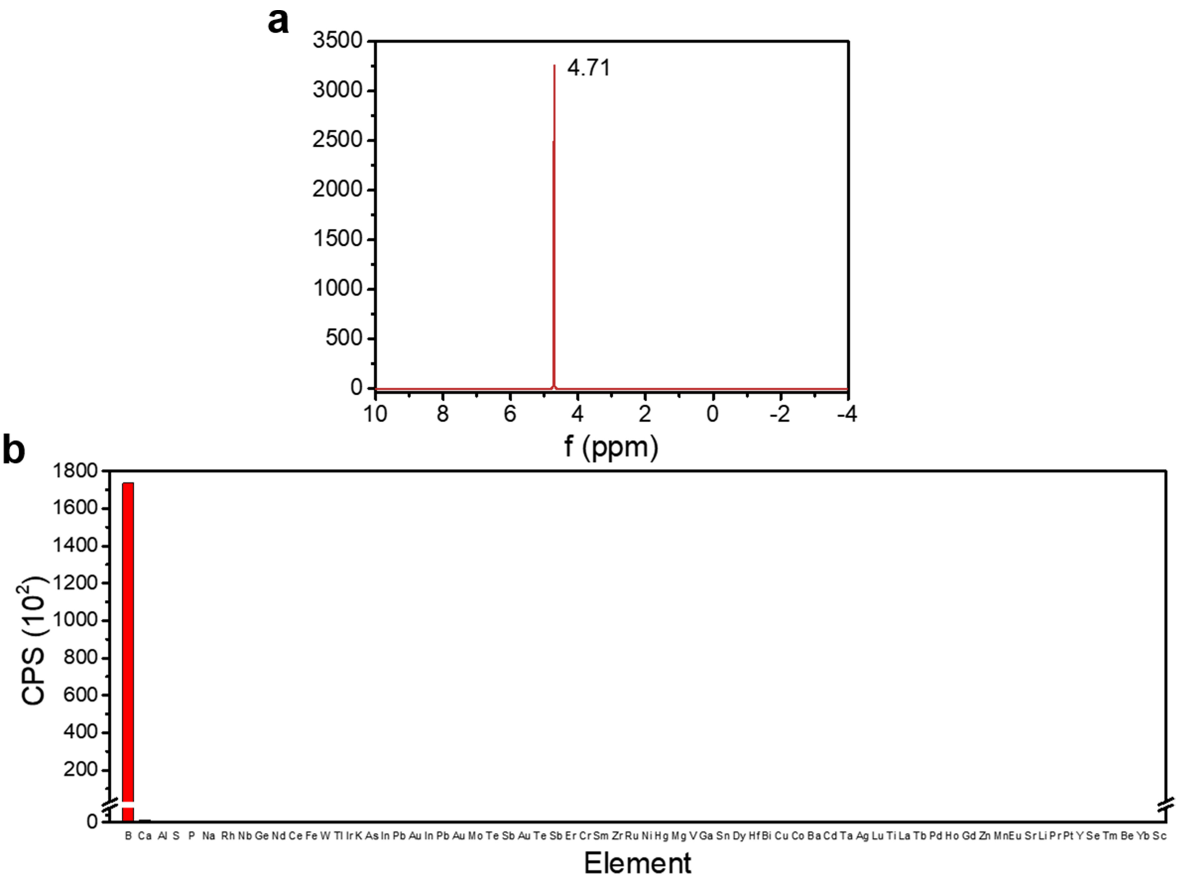

Figure S1. (a) ^{1}H NMR and (b) ICP-MS spectra of recrystallized BA.

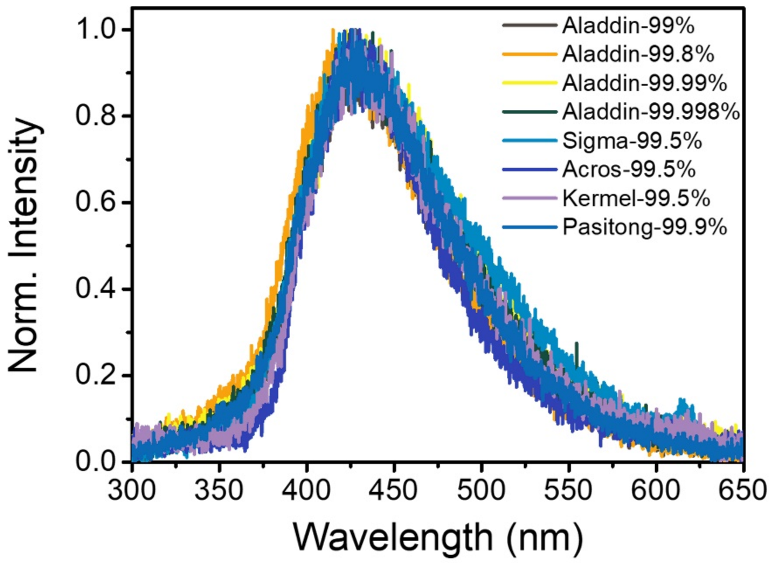

Figure S2. Normalized delayed PL emission spectra of BA from different manufactures with different purity. All the spectra were recorded under the excitation of 260 nm.

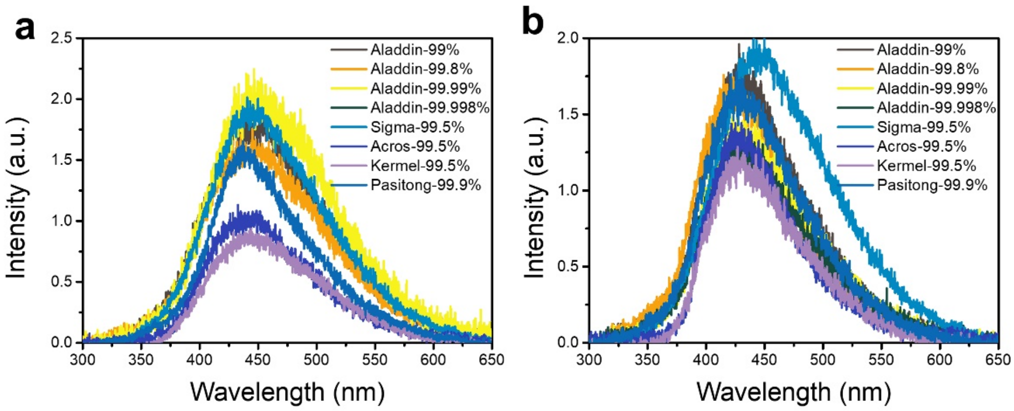

Figure S3. Delayed PL emission spectra of BA from different manufactures with different purity (a) before and (b) after recrystallization. All the spectra were recorded under the excitation of 260 nm.

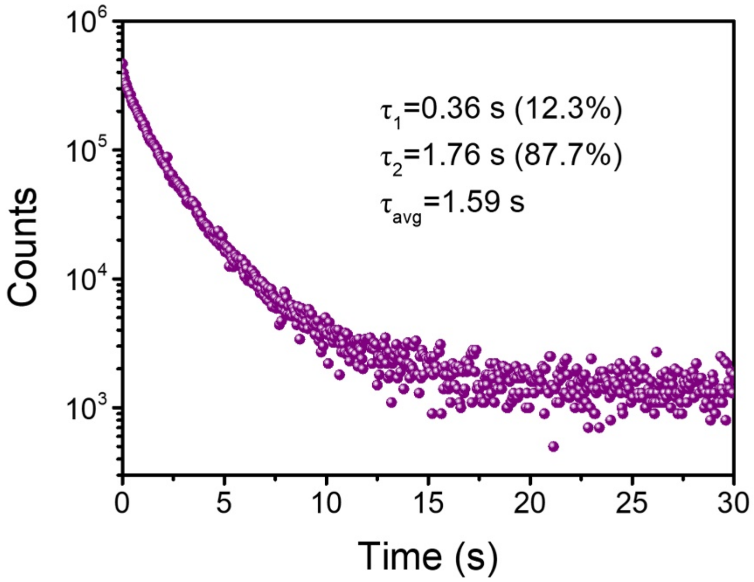

Figure S4. Emission decay curve of heated BA, which was recorded under the excitation of 260 nm and emission of 430 nm.

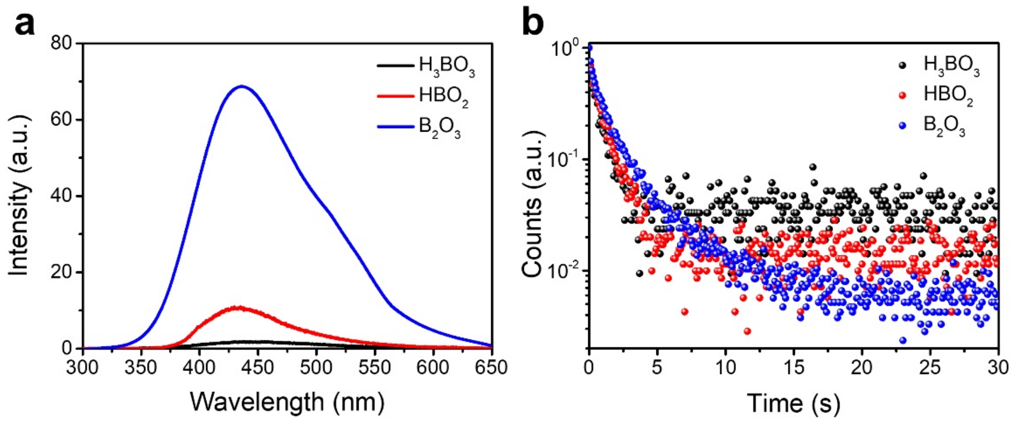

Figure S5. (a) Delayed PL emission spectra and (b) emission decay curves of H_3BO_3, HBO_2 and B_2O_3. All the spectra and curves were recorded under the excitation of 260 nm, and the decay curves were detected at 430 nm.

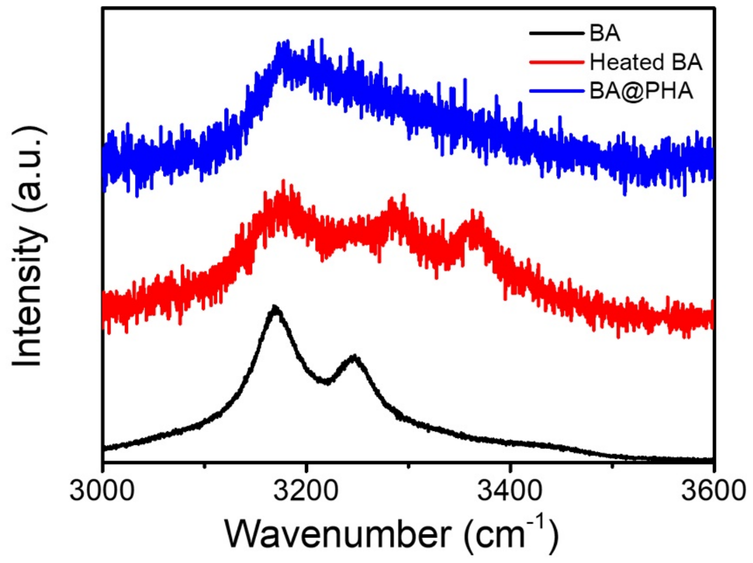

Figure S6. Raman spectra of BA, Heated BA and BA@PHA.

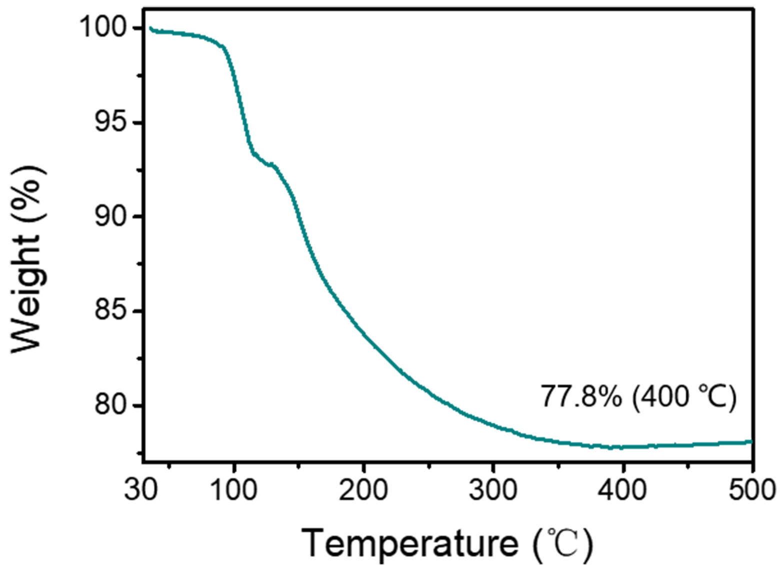

Figure S7. TGA curve of heated BA.

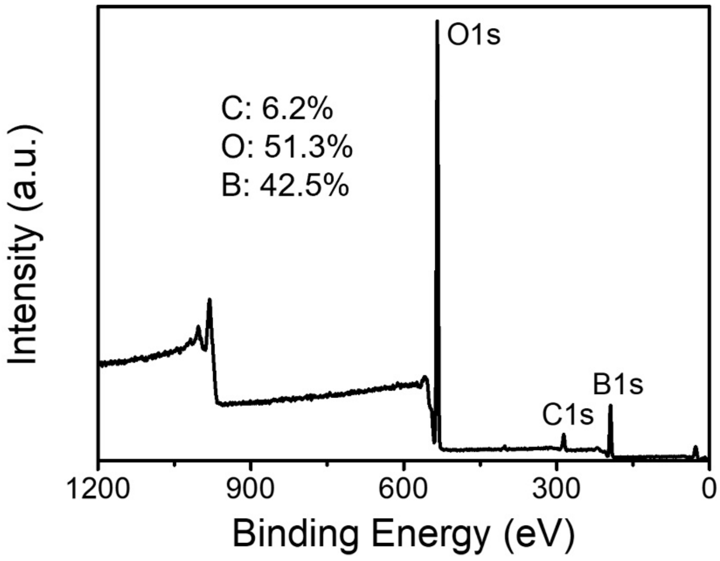

Figure S8. Full scan XPS spectrum of BA@PHA, with the content of C, O and B showing as inset.

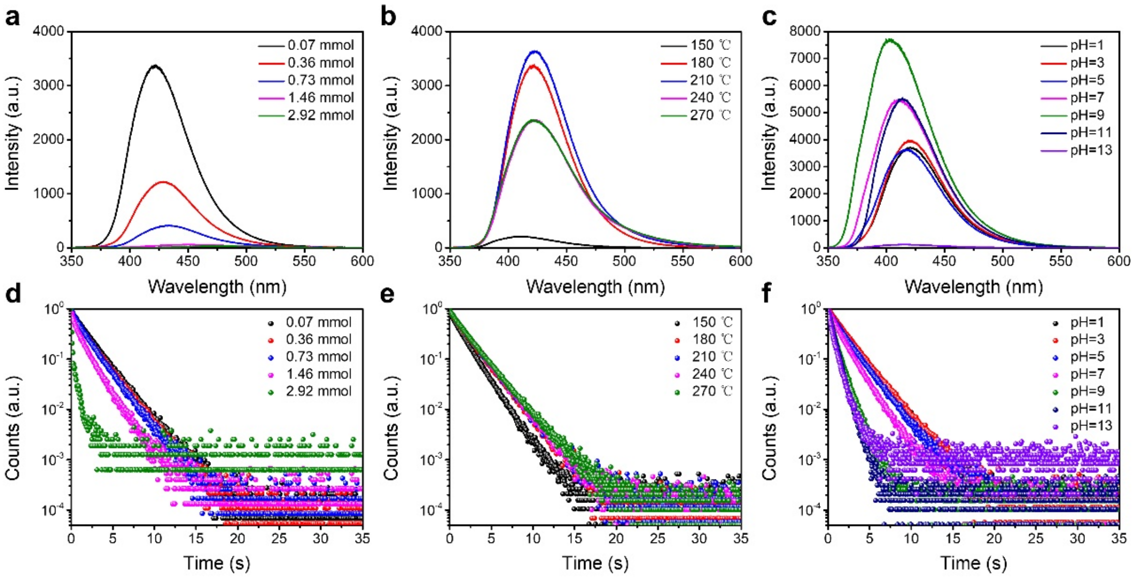

Figure S9. Delayed PL emission spectra of BA@PHA produced (a) by loading different amount of PHA, (b) at different heating temperature, and (c) under different pH, with corresponding emission decay curves shown in (d), (e) and (f). All the spectra and curves were recorded under the excitation of 280 nm, the emission curves were detected at 430 nm.

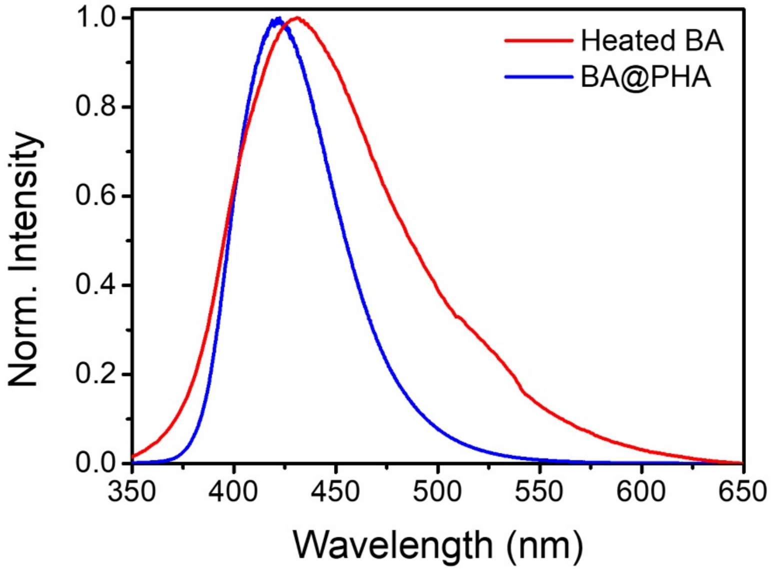

Figure S10. Normalized delayed PL emission spectra of Heated BA and BA@PHA, excited at 260 nm.

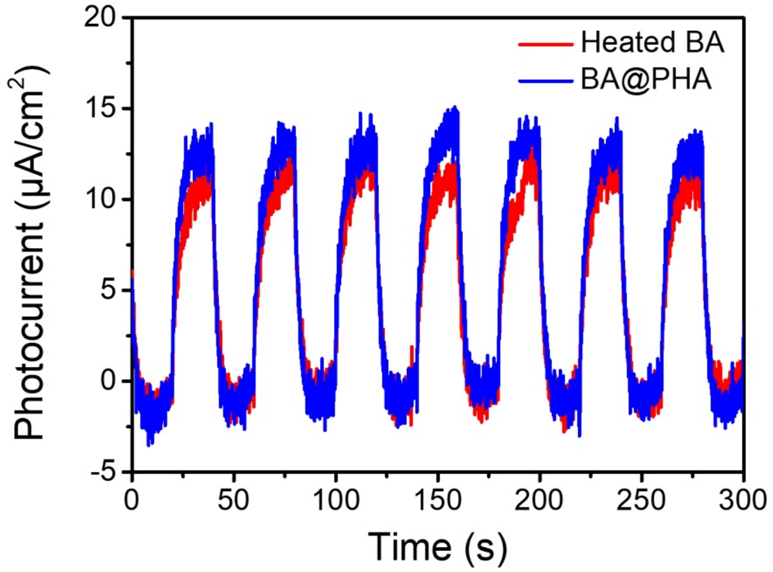

Figure S11. Photocurrent curves of heated BA (red line) and BA@PHA (blue line).

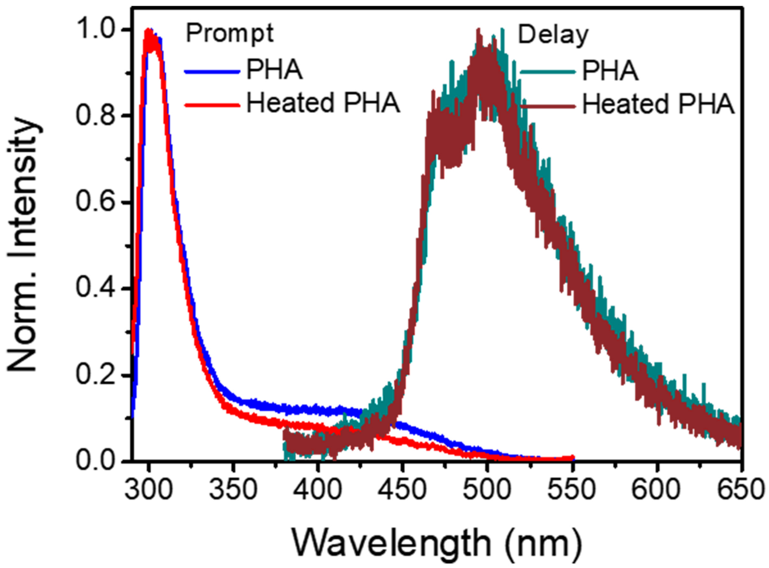

Figure S12. Normalized prompt and delayed PL emission spectra of before and after heat treatment, excited at 280 and 360 nm, respectively.

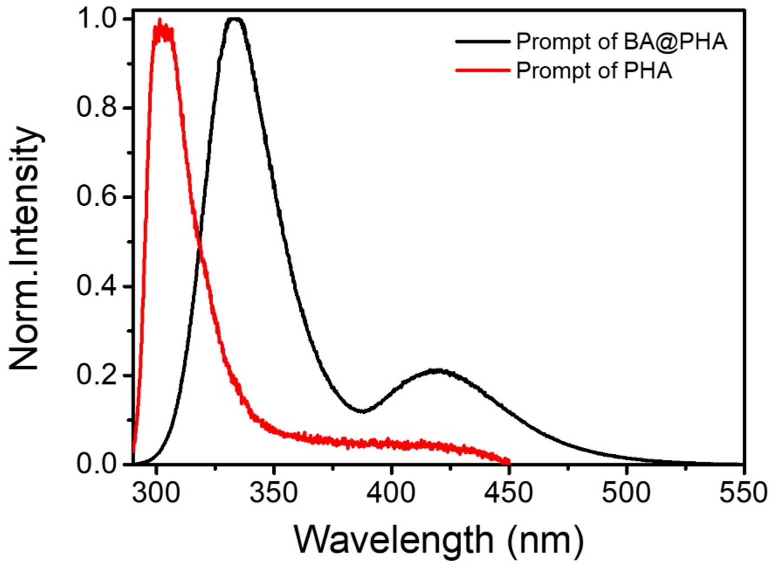

Figure S13. Normalized prompt PL emission spectra of BA@PHA and PHA, excited at 280 nm.

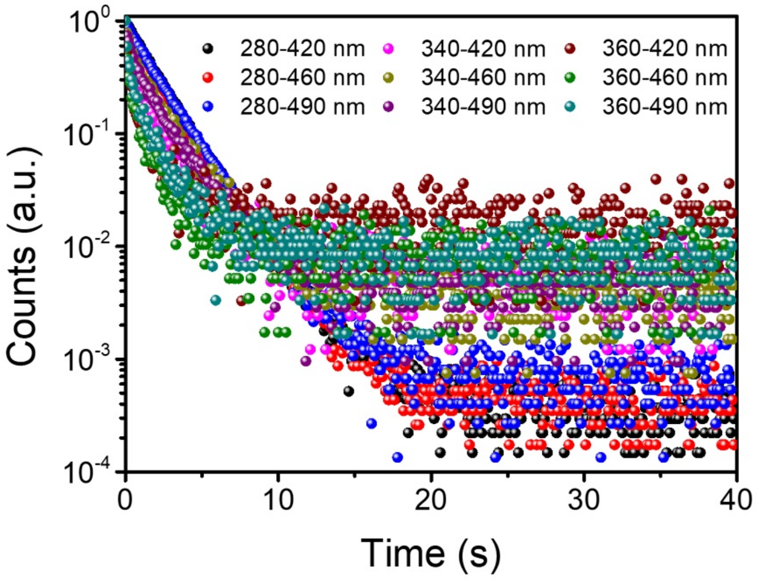

Figure S14. Emission decay curves of BA@PHA, recorded at different excitation and emission wavelengths.

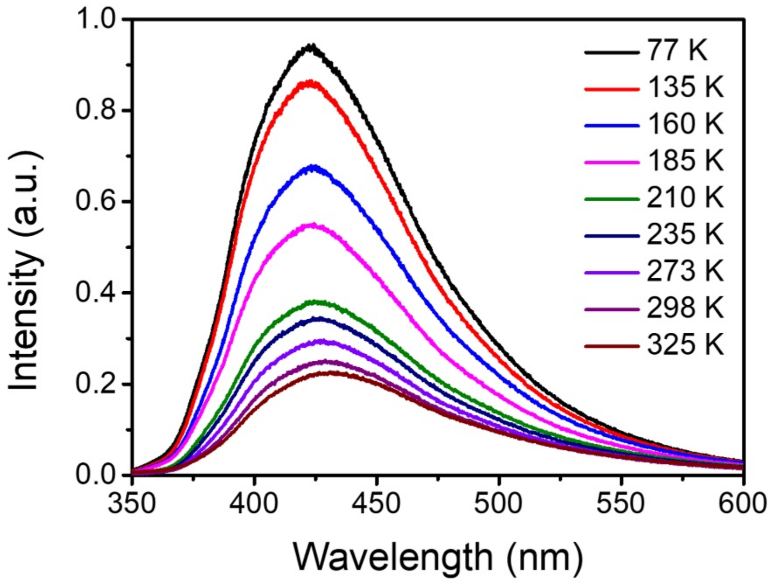

Figure S15. Delayed PL emission spectra of heated BA recorded at different temperature, as indicated on the frame. All the spectra were recorded under the excitation of 260 nm.

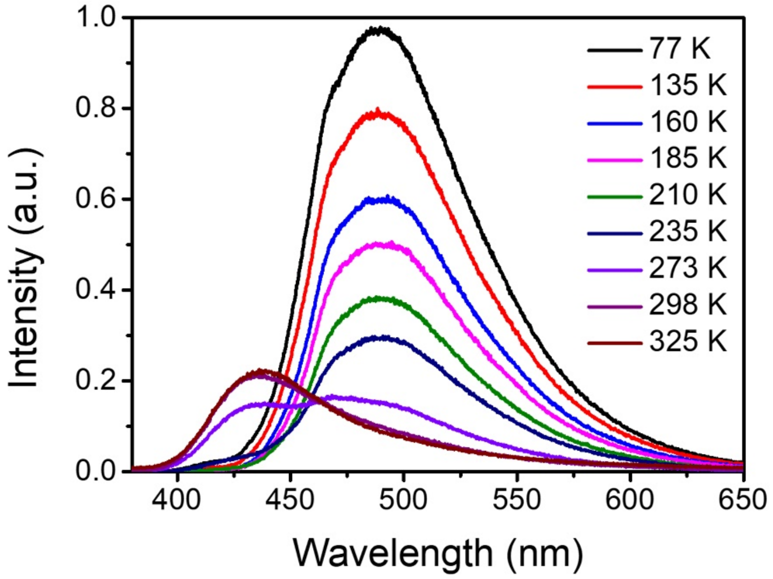

Figure S16. Delayed PL emission spectra of PHA recorded at different temperature, as indicated on the frame. All the spectra were recorded under the excitation of 360 nm.

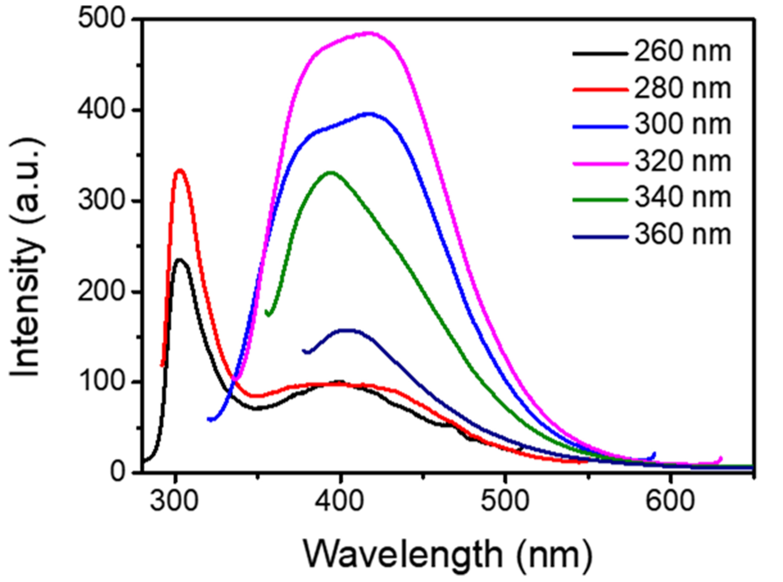

Figure S17. Prompt PL emission spectra of PHA recorded at different excitation wavelength.

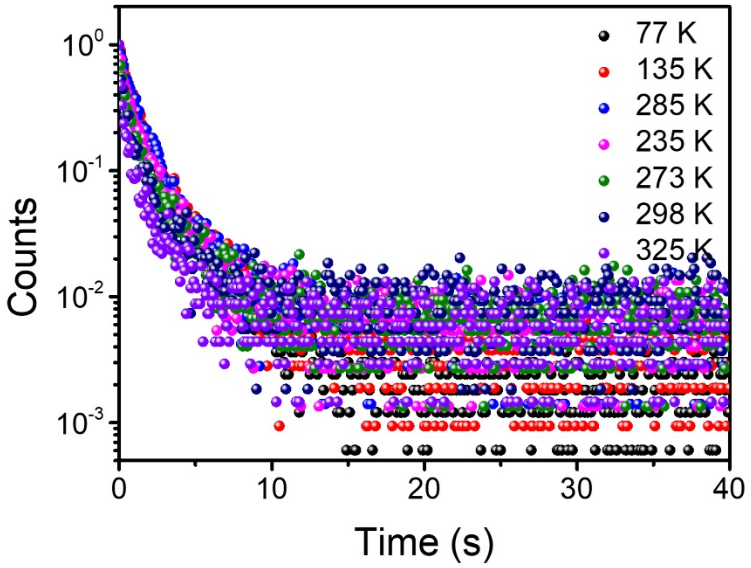

Figure S18. Emission decay curves of BA@PHA detected at different temperature. All the curves were recorded at 48 nm, under the excitation of 360 nm.

3. Tables

Table S1. Peak maximum of the delayed PL spectra and lifetimes of BA from different manufactures and with different purities, before and after recrystallization.

Brands	Purity	Raw Materials		Recrystallization	
		λ_{Em} (nm)	τ (s)	λ_{Em} (nm)	τ (s)
Aladdin	99%	437.8	0.83	432.6	0.86
Aladdin	99.8%	445.2	0.74	423.0	0.77
Aladdin	99.99%	437.0	1.25	431.4	1.01
Aladdin	99.998%	440.6	0.88	426.4	0.92
Sigma	99.5%	445.6	0.94	427.4	0.74
Acros	99.5%	441.8	0.73	430.2	1.01
Kermel	99.5%	442.4	0.67	427.8	0.96
Pasitong	99.5%	437.2	1.04	430.4	0.95

Table S2. Emission lifetimes (τ_{1-2}, s) and fractions of the emission intensity (f_{1-2}, %) obtained from the fitting of experimental emission decay data by two-exponential functions of H_3BO_3, HBO_2, B_2O_3, from which the average emission lifetimes τ_{avg} (s) were calculated. All the curves were detected at 430 nm, under the excitation of 260 nm.

Name	τ_1 (f_1)	τ_2 (f_2)	τ_{avg}
H_3BO_3	0.21 (17.5)	0.96 (82.5)	0.83
HBO_2	0.22 (10.8)	1.10 (89.2)	1.00
B_2O_3	2.11 (80.2)	0.45 (19.8)	1.78

Table S3. Emission lifetimes ($\tau_{1\text{-}2}$) and fractions of the emission intensity ($f_{1\text{-}2}$, %) obtained from the fitting of experimental emission decay data of BA@PHA, recorded at different wavelengths, by two-exponential functions, from which the average emission lifetimes τ_{avg} were calculated. All the curves were under the excitation of 280 nm.

λ_{Em} (nm)	τ_1 (f_1)	τ_2 (f_2)	τ_{avg}
334 nm	1.96 ns (100)	—	1.96 ns
420 nm	0.83 s (6.4)	2.09 s (93.6)	2.01 s

Table S4. Emission lifetimes ($\tau_{1\text{-}2}$, s) and fractions of the emission intensity ($f_{1\text{-}2}$, %) obtained from the fitting of experimental emission decay data by two-exponential functions of BA@PHA, produced by loading different amount of PHA, from which the average emission lifetimes τ_{avg} (s) were calculated. All the curves were detected at 420 nm, under the excitation of 280 nm.

Amount (mmol)	τ_1 (f_1)	τ_2 (f_2)	τ_{avg}
0.07	0.83 (6.4)	2.09 (93.6)	2.01
0.36	0.89 (8.0)	2.06 (92.0)	1.97
0.73	0.44 (7.5)	1.95 (92.5)	1.84
1.46	0.34 (16.7)	1.61 (83.3)	1.40
2.92	0.25 (18.5)	1.21 (81.5)	1.03

Table S5. Emission lifetimes ($\tau_{1\text{-}2}$, s) and fractions of the emission intensity ($f_{1\text{-}2}$,%) obtained from the fitting of experimental emission decay data by two-exponential functions of BA@PHA, produced by heating under different temperature, from which the average emission lifetimes τ_{avg} (s) were calculated. All the curves were detected at 420 nm, under the excitation of 280 nm.

Temperature (°C)	τ_1 (f_1)	τ_2 (f_2)	τ_{avg}
150	1.66 (95.6)	0.59 (4.4)	1.62
180	0.83 (6.4)	2.09 (93.6)	2.01
210	1.12 (10.3)	2.14 (88.7)	2.03
240	0.84 (0.6)	2.08 (99.4)	2.08
270	2.17 (92.6)	0.92 (7.4)	2.08

Table S6. Emission lifetimes ($\tau_{1\text{-}2}$, s) and fractions of the emission intensity ($f_{1\text{-}2}$, %) obtained from the fitting of experimental emission decay data by two-exponential functions of BA@PHA, produced at different reaction pH values, from which the average emission lifetimes τ_{avg} (s) were calculated. All the curves were detected at 420 nm, under the excitation of 280 nm.

pH	τ_1 (f_1)	τ_2 (f_2)	τ_{avg}
1	0.88 (9.2)	2.07 (90.8)	1.96
3	0.90 (9.5)	2.07 (90.6)	1.96
5	0.98 (14.0)	2.03 (86.0)	1.88
7	0.80 (27.6)	1.60 (72.4)	1.38
9	0.41 (36.7)	0.92 (63.3)	0.73
11	0.37 (26.3)	0.73 (73.7)	0.64
13	0.18 (16.7)	0.92 (83.3)	0.57

Table S7. Phosphorescence lifetimes (τ_{1-2}) and fractions of the emission intensity (f_{1-2}, %) obtained from the fitting of experimental phosphorescence decay data by two-exponential functions of BA@OHA, BA@MHA and BA@PHA, from which the average emission lifetimes τ_{avg} were calculated. All the curves were under the excitation of 280 nm.

**Name**	**λ_{Em} (nm)**	**τ_1 (f_1)**	**τ_2 (f_2)**	**τ_{avg}**
BA@OHA	400	0.006 s (19.2)	0.90 s (80.8)	0.73 s
BA@MHA	437	0.81 s (20.0)	2.11 s (80.0)	1.85 s
BA@PHA	420	0.83 s (6.4)	2.09 s (93.6)	2.01 s

Table S8. Emission lifetimes (τ_{1-2}, s) and fractions of the emission intensity (f_{1-2}, %) obtained from the fitting of experimental phosphorescence decay data by two-exponential functions of BA@PHA at different excitation (λ_{Ex}, nm) and emission wavelengths (λ_{Em}, nm), from which the average emission lifetimes τ_{avg} (s) were calculated.

**λ_{Ex}**	**λ_{Em}**	**τ_1(f_1)**	**τ_2 (f_2)**	**τ_{avg}**
280	420	0.83 (6.4)	2.09 (93.6)	2.01
	460	2.03 (98.0)	0.52 (2.0)	2.01
	490	2.08 (95.1)	0.82 (4.9)	2.01
340	420	0.24 (16.0)	1.79 (84.0)	1.54
	460	1.98 (91.7)	0.25 (8.3)	1.84
	490	0.25 (9.5)	1.86 (90.5)	1.70
360	420	1.13 (77.8)	0.13 (22.2)	0.91
	460	1.05 (79.8)	0.11 (20.2)	0.86
	490	0.14 (16.1)	1.32 (83.9)	1.13

Table S9. Emission QY of BA@PHA at different excitation wavelengths (λ_{Ex}, nm).

λ_{Ex}	Total (%)	Afterglow (%)
290	83.78	32.08
300	60.94	23.10
310	54.11	21.57
320	34.85	16.37
330	19.10	13.48

Table S10. Phosphorescence lifetimes (τ_{1-2}, s) and fractions of the emission intensity (f_{1-2},%) obtained from the fitting of experimental phosphorescence decay data by two-exponential functions of BA@PHA at different test temperature, from which the average emission lifetimes τ_{avg} (s) were calculated. All the curves were detected at 480 nm, under the excitation of 360 nm.

Temperature (K)	τ_1 (f_1)	τ_2 (f_2)	τ_{avg}
77	1.84 (73.2)	0.51 (26.8)	1.48
135	0.55 (25.6)	1.94 (74.4)	1.58
185	1.93 (73.8)	0.53 (26.2)	1.56
235	0.30 (16.9)	1.63 (83.1)	1.40
273	1.47 (81.4)	0.22 (18.6)	1.24
298	1.35 (79.7)	0.18 (20.3)	1.12
325	0.95 (84.4)	0.08 (15.6)	0.82
